# Effect of Blanching and Boiling on the Secondary Metabolism of Cultivated Cardoon Stalks: A Case Study of the Tuscany Region (Italy)

**DOI:** 10.3390/metabo12080728

**Published:** 2022-08-06

**Authors:** Costanza Ceccanti, Luigi De Bellis, Lucia Guidi, Carmine Negro, Alberto Pardossi, Luca Incrocci

**Affiliations:** 1 Department of Agriculture, Food and Environment, University of Pisa, 56124 Pisa, Italy; 2 Interdepartmental Research Center, Nutrafood, “Nutraceuticals and Food for Health”, University of Pisa, 56124 Pisa, Italy; 3 Department of Biological and Environmental Sciences and Technologies, University of Salento, 73100 Lecce, Italy

**Keywords:** antioxidant activity, blanching, *Cynara cardunculus*, cynaropicrin, heat treatment, phenolics

## Abstract

Cardoon (*C. cardunculus* var. *altilis* DC) is commonly cultivated in the Mediterranean area to produce stalks that are consumed once cooked. Before cooking, stalks are usually subjected to blanching, which means they are exposed to darkness for a few weeks. The present work analyzed the effect of field blanching carried out for 40 days in different ways (burying the stalks under soil or covering them with plastic sheet) on the total phenolic content (TPC), phenolic profile, cynaropicrin content (a bitter compound), and antioxidant activity (AA) of two cardoon cultivars. The nutraceutical quality of blanched cardoons was also investigated following boiling. The phenolic profile revealed a higher number of compounds in blanched stalks than in raw ones. The cynaropicrin content decreased in both cultivars after blanching, indicating a sensitivity to dark conditions and the effectiveness of blanching method in reducing its bitterness. The data presented contribute to improving the knowledge about the effect of blanching and boiling on the quality of cardoon stalks.

## 1. Introduction

*Cynara cardunculus* L. is a species belonging to the Asteraceae family and includes artichoke (*C. cardunculus* var. *scolymus* (L.) Fiori), cultivated cardoon (*C. cardunculus* var. *altilis* DC), and wild cardoon (*C. cardunculus* var. *sylvestris* (Lamk) Fiori) [1]. These species have a perennial biological cycle with a period of vegetative growth occurring from autumn to spring, the period with the most abundant rain in the Mediterranean area, while the ripening of seeds occurs in the summer [2].

Cultivated cardoon is popular in the Mediterranean region, mainly in Spain, France, Italy, Greece, and southern Portugal [3,4]. This crop is used to produce fleshy stalks that are consumed as a traditional dish once boiled, fried, sautéed, or baked [4,5]. Cardoon stalks are usually subjected to blanching, which exposes stalks to dark conditions for about a few weeks in the field. This process is performed in several different ways according to the labor requirement and cost-effectiveness. Blanching is carried out to reduce the bitterness and to accentuate the flavor and tenderness of cardoon stalks [5]. Indeed, this vegetable is characterized by a strong bitter taste due to the high content of cynaropicrin (56–83%), followed by geosheimin (2–18%) and cuanaratriol (4–22%) [6]. Cynaropicrin is a sesquiterpene with marked antioxidant and medicinal properties such as antiproliferative, antifeedant, and anti-inflammatory properties [7,8]. Moreover, the antioxidant activity (AA) of cultivated cardoon stalks is also due to the high content of flavonoids and phenolic acids. However, few works have reported the phenolic profile of cardoon stalks, underlining their richness in caffeoylquinic acid derivatives and the glycosidic forms of apigenin and luteolin [3,4,5,9].

Moreover, to the best of our knowledge, only Pinelli et al. [5] has studied the effect of blanching on the content of bioactive compounds in cardoon stalks, revealing a decrease in polyphenols after blanching, without any specification of the blanching method [5]. Likewise, only two recent articles analyzed the effect of cooking and, consequently, of heat treatment, on the bioactive compounds and AA of cardoon stalks [4,10]. In addition, Huarte et al. [4] revealed that a blanching treatment (98 °C for 30 s) increased the content of caffeoylquinic acids in cardoon stalks without any significant effect on the AA when determined using a 2,2′-diphenyl-1-picrylhydrazyl radical (DPPH) assay. In comparison, the frying treatment decreased the AA, notwithstanding an increase in the flavonoid content [4]. Similarly, Juániz et al. [10] found an increase in the concentration of phenolic compounds in cardoon stalks once fried or grilled.

The aim of the present work was the assessment of the effect of different blanching methods in the field and boiling on the phenolic and cynaropicrin content, the phenolic profile, and the AA of cardoon stalks grown in a field in the Mediterranean area. For the first time, both blanching and heat treatment on cardoon were analyzed in the same paper, presenting some ideas of the nutraceutical quality of the product utilized by the consumer.

## 2. Materials and Methods

### 2.1. Plant Material

Two experiments were carried out with different cultivars of *C. cardunculus* var. *altilis*, Pieno Inerme Lucchese (PIL) and Plain Blanc Inerme (PBI), cultivated in a field in Lucca (Italy) and in Peccioli (Pisa, Italy), respectively. These were either subjected or not subjected to stalk blanching, which was performed using two different methods: stalks were either buried under loose soil (PIL) or wrapped with a 0.5 mm-thick white and black plastic sheet made of low-density polyethylene (LDPE; Jolly Plastic, Larciano, PT) (PBI). In both experiments, blanching was applied for 40 days before the harvest, which took place on 29 January 2021.

Three samples, each consisting of blanched or unblanched stalks collected from separate plants, were collected from both fields, and three sub-samples were immediately frozen in liquid nitrogen and stored at –80 °C until the biochemical analysis. Another three subsamples were frozen in liquid nitrogen and then lyophilized and stored at –80 °C for the chromatographic identification and quantification of cynaropicrin and phenolic compounds. Three sub-samples were also taken and dried in a ventilated oven (Memmert GmbH Co., KG Universal Oven UN30, Schwabach, Germany) at 105 °C until the sub-sample reached a constant weight for the determination of the moisture content.

### 2.2. Boiling Treatment

Blanched stalks were washed with distilled water and cut into rectangular homogeneous pieces (2 cm × 2 cm approx.), manually mixed and divided into three portions (150 g of each one), and then boiled in 600 mL of distilled water for 30 min. Boiled stalks were sampled for laboratory determinations as previously described.

### 2.3. Total Phenolic Content (TPC) Assay

The TPC was determined following the method in Dewanto et al. [11]. The oxidation of phenolic compounds along with the reduction in the metals present in the solution of phosphomolybdate–phosphotungstate of the Folin–Ciocalteu reagent and the consequent color blue were measured at 760 nm with a spectrophotometer (Ultrospec 2100 Pro, GE Healthcare Ltd., Chalfont, Buckinghamshire, UK). The TPC was expressed as mg gallic acid equivalents (GAE) per g dry weight (DW).

### 2.4. Cynaropicrin and Phenolic Compound Extraction

The extraction of cynaropicrin and phenolic compounds was performed according to Zhou et al. [12] using methanol at a ratio of 1:20 (*w*/*v*). An amount of lyophilized cardoon stalk was homogenized in liquid nitrogen and in a 75% (*v*/*v*) aqueous solution of methanol mixed with 0.1% formic acid. Samples were sonicated twice in a water bath sonicator (VWR, Leuven, Belgium) for 30 min and then agitated for 20 min. The supernatant was filtered at 0.2 μm with a polytetrafluoroethylene filter before UHPLC analysis.

### 2.5. Cynaropicrin and Phenolic Characterization

Phenolic characterization was performed by an Agilent 1200 Liquid Chromatography system (Agilent Technologies, Palo Alto, CA, USA) equipped with a standard autosampler as reported by Negro et al. [13]. The UHPLC column was an Agilent Extended C18 (1.8 µm, 2.1 × 50 mm). Separation was carried out at 40 °C with a gradient elution program at a flowrate of 0.5 mL min^−1^. The mobile phases consisted of water plus 0.1% formic acid (A) and acetonitrile (B). The following multistep linear gradient was applied: 0–2 min, 1% B; 13 min, 25% B; 19 min, 40% B; 21 min, 90%B.

The UHPLC system was coupled to an Agilent 6320 TOF mass spectrometer equipped with a dual electrospray ionization (ESI) interface (Agilent Technologies, Palo Alto, CA, USA), and a calibration solution containing the internal reference mass was introduced. The system operated on a negative-ion mode to obtain the phenolic characterization and on a positive-ion mode to obtain cynaropicrin characterization and quantification. Accurate measurements of the mass corresponding to each total ionic current (TIC) peak were obtained with a pump (Agilent G1310B) with a low flow (20 µL min^−1^) of a calibration solution containing internal reference masses at *m*/*z* 112.9856, 301.9981, 601.9790, and 1033.9881 for negative ions and 121.050873, 149.023320, 322.048121, and 922.009798 for positive ions, and using a dual nebulizer ESI source. A cynaropicrin standard was solubilized in a solution of methanol–water at 75:25 (*v*/*v*) and diluted with water–formic acid at 99.9:0.1 (*v*/*v*) and at a final concentration of 0.5–10 μg mL^−1^. 

### 2.6. Antioxidant Activity (AA) Assay

The AA was measured as reported by Brand-Williams et al. [14]. An amount (10 μL) of phenolic extract was mixed with 990 μL of a methanolic solution of 3.12 × 10^−1^ M DPPH (*w*/*v*) and left alone for 30 min for the DPPH radical to react with the antioxidant compounds present in the samples. The change in color (from violet to pink) of DPPH due to the reduction in this reagent by the antioxidant compounds in the extract solution was measured at 515 nm against a blank solution (with no extracts) with the same spectrophotometer used for the determination of the TPC. The AA was expressed as mg Trolox equivalents (TE) per g DW.

### 2.7. Statistical Analysis

Data from each experiment were subjected to a one-way ANOVA, and the means (± standard error) were separated using Tukey’s test. All statistical analyses were conducted using GraphPad (GraphPad, La Jolla, CA, USA).

## 3. Results and Discussion

### 3.1. Effect of Blanching

**Figure 1:**
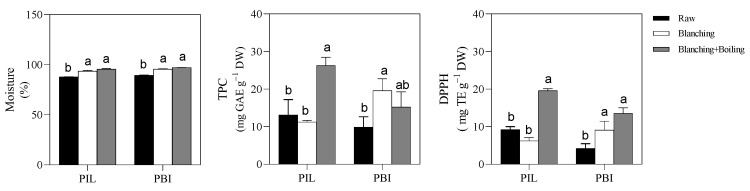
Moisture, total phenolic content (TPC), and antioxidant activity (AA) of stalks of two cardoon cultivars (*Cynara cardunculus* var. *altilis* DC cv. Pieno Inerme Lucchese, PIL, and *Cynara cardunculus* var. *altilis* DC cv. Plain Blanc Inerme; PBI) grown in the field. Raw, blanched, and blanched+boiled cardoon stalks are represented by closed, open, and gray bars, respectively. For each cultivar separately, means (±SE; *n* = 3) indicated by different letters differ significantly (*p* ≤ 0.05). GAE: gallic acid equivalents; TE: Trolox equivalents; DW: dry weight.

Figure 1 shows the moisture content, the TPC, and AA of unblanched (control), blanched, and boiled and blanched stalks of PIL and PBI.

In both cultivars, blanching significantly increased the moisture content of stalks. In PBI, the TPC and AA of blanched stalks were greater compared with the controls, whilst in PIL, no significant differences were found in the TPC and AA. The TPC values of both PIL and PBI yielded very similar results to those reported by Huarte et al. [4] for raw *C. cardunculus* var. *altilis* stalks, and the AA values found by these authors yielded similar results to the PIL results (36 μmol TE g^−1^ DW) but were higher than those reported for raw PBI stalks (17 μmol TE g^−1^ DW). Indeed, Huarte et al. [4] reported a TPC value of 8.28 ± 0.03 mg GAE g^−1^ DW and an AA value of 39.5 ± 4.72 μmol TE g^−1^ DW. The AA values of PBI accorded with the results of Huarte et al. [4] once subjected to the blanching process (about 36 μmol TE g^−1^ DW), likely because the Spanish authors analyzed cardoon stalks that were purchased in a local market, which had surely already been subjected to the blanching process. We could hypothesize that the cardoon stalks utilized in Huarte et al.’s [4] experiment were subjected to blanching with a plastic sheet, given the similarity of the TPC and AA results with our results for blanched PBI stalks. Although no information about different blanching processes is present in the literature, this increase in the nutraceutical quality of PBI after the blanching process could likely be due to the increase in temperature and relative humidity caused by the plastic wrapping [15,16], as suggested by the results of the UHPLC/DAD/TOF analysis reported in Table 1. Indeed, blanched PBI stalks yielded the richest content in different phenolic compounds; in these stalks, 20 phenolic compounds were identified, 11 of which were not found in the controls.

Twenty-nine phenolic compounds belonging to the phenolic acid, catechol, and flavonoid classes were found in *C. carduculus* var. *altilis* samples, and twenty-five of these were also identified. Only the quinic acid isomer was found in all samples (independently of blanching). Five phenolic compounds were found in the control PIL stalks and six in the blanched stalks, while only one compound was found in both unblanched and blanched samples. The presence of flavonoids and caffeoylquinic acids in *C. cardunculus* was confirmed in other species or/and varieties of the genus *Cynara* by other authors [4,5,9].

Dihydroxybenzene, quinic acid II, p-cumaroylquinic acid, and kaempferol in PIL samples, and monocaffeoylquinic acid II and p-cumaroylquinic acid in PBI samples, were no longer detectable after blanching (Table 1). These findings agree with those of Pinelli et al. [5], who reported the disappearance of some phenolic compounds in cardoon stalks after blanching.

In PIL, the content of the main phenolics was under the detection limit or higher in control samples than in blanched samples (Table 2). At the same time, new phenolic compounds were found in blanched samples compared with the compounds identified in controls.

We hypothesized that the influence of the different environmental conditions underground in PIL and, especially, under a plastic sheet in PBI, could accelerate the plant’s defense responses, resulting in an accumulation of new phenolic compounds. This hypothesis was confirmed by Dawidowicz and Typek [23], although they analyzed a different matrix (coffee beans). Indeed, they observed the degradation of chlorogenic acid isomers by heat treatment that caused their transformation to quinic acid and chlorogenic acid lactones, changing the ratio between chlorogenic acid isomers and mono- and di-caffeoylquinic acid isomers.

**Figure 2:**
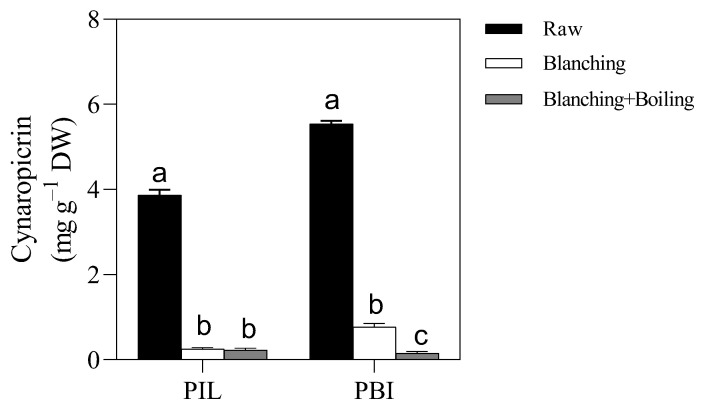
Content of cynaropicrin in stalks of cardoon (*Cynara cardunculus* var. *altilis* DC cv. Pieno Inerme Lucchese; PIL and *Cynara cardunculus* var. *altilis* DC cv. Plain Blanc Inerme; PBI). Raw, blanched, and blanched+boiled cardoon stalks are represented by closed, open, and gray bars, respectively. For each cultivar separately, means (±SE; *n* = 3) indicated by different letters differ significantly (*p* ≤ 0.05).

Moreover, we found a lower cynaropicrin content in blanched stalks than in controls in both PLI and PBI (Figure 2). Scavo et al. [24] found a lower cynaropicrin content in wild cardoon stalks grown under shade in winter (about 25 mg L^−1^) as compared with cardoon stalks grown under full light in winter (about 45 mg L^−1^). These authors hypothesized that this was due to a reduction in the terpenoid biosynthesis. Taking into account the darkness induced by the blanching, this explanation could also explain our results.

### 3.2. Effect of Boiling

The boiling treatment did not affect the TPC of the stalks of either PBI, whilst it increased the TPC of PIL stalks and the AA of PIL and PBI stalks compared with the controls (Figure 1).

The pattern of the AA in boiled PIL and PBI stalks agreed with the findings of Juániz et al. [10]. These authors reported a high increase in AA when cardoon was subjected to 150 °C for 10 min and then fried in oil at 110 °C for 5 min (90 μmol TE g^−1^ DW) compared to raw samples (about 30 μmol TE g^−1^ DW), which showed a small but non-significant increase in AA when cardoon was fried in sunflower oil (about 35 μmol TE g^−1^ DW), and a decrease when they were fried in olive oil (about 20 μmol TE g^−1^ DW). These authors ascribed the high increase in the AA of the cardoon stalks subjected to temperatures between 110 and 150 °C to the destruction of cell walls and subcellular compartments, which favored the release of bioactive compounds from internal compartments of plant cells at the cooking mean, independently of the plant matrix under investigation [10]. Conversely, Martini et al. [25] suggested that the food matrix played an important role in the retention of phenolic compounds after heat treatment. Nevertheless, no studies on the use of boiling for cardoon stalks have been reported in the literature.

The UHPLC/DAD/TOF analysis showed the presence of a higher number of phenolic compounds in boiled stalks when compared to the raw ones (Table 1). Forty-three phenolic compounds belonging mainly to the class of phenolic acids were identified, whilst seven compounds remained unknown. The most representative compounds were the quinic and caffeoylquinic acids and their derivatives. Among the latter, the monocaffeoylquinic acid, dicaffeoylquinic acid, and monocaffeoylquinic acid dimer were the most abundant in boiled PIL stalks, whilst the dicaffeoylquinic acid was the most representative compound in PBI stalks (Table 2). Other authors showed the retention of caffeic acid derivatives after heat treatment in cardoon stalks but also in different plant matrices, such as eggplant, confirming the heat resistance of this class of compounds [10,25].

The blanching and boiling of cardoon stalks are necessary to reduce their bitterness, which is due to the content of some sesquiterpenes, especially cynaropicrin, and to increase their tenderness.

No significant effect of the boiling treatment was found on the cynaropicrin content of blanched, raw PIL stalks, whilst a lower cynaropicrin content was observed in the boiled PBI stalks, likely due to a higher starting content of cynaropicrin in PBI stalks than in PIL stalks (Figure 2).

## 4. Conclusions

The present work analyzed the effect of field blanching and boiling on some biochemical attributes of cardoon stalks grown in the Mediterranean area. As expected, blanching led to a decrease in the cynaropicrin content while inducing the synthesis of some phenolic compounds that were not detected in unblanched stalks. Similarly, the boiling treatment for blanched stalks reduced their cynaropicrin content and increased the antioxidant capacity and the diversity of phenolic acids as compared to non-boiled blanched stalks. This study was a first step towards the assessment of the nutraceutical quality of processed cardoon stalks, as there was little information in the literature about this vegetable. However, more investigations are needed for a deeper understanding of the effect of field blanching and boiling on the biochemical characteristics of cardoon stalks.

## Figures and Tables

**Table 1:** Identification of phenolic compounds, [M-H]^-^, by UHPLC/DAD/TOF analysis of stalks of cardoon (*Cynara cardunculus* var. *altilis* DC cv. Pieno Inerme Lucchese; PIL and *Cynara cardunculus* var. *altilis* DC cv. Plain Blanc Inerme; PBI) cultivated in the field. The symbol (+) indicates the presence of that phenolic compound in the sample. MW: molecular weight.

N^o^	Name	Formula [M-H]^-^	Exp. MW [M-H]^-^	Calculated MW [M-H]^-^	∆, ppm	Score	Unbl. PIL	Bl. PIL	Boil. PIL	Unbl. PBI	Bl. PBI	Boil. PBI	Refs
1	Quinic acid I	C_7_H_12_O_6_	191.0575	191.0561	−7.15	87.28	+	+	+	+	+		[17]
2	Unknown	C_13_H_17_O_12_	365.0737	365.0725	−3.15	73.66						+	-
3	Poly-galacturonic acid methyl ester	C_20_H_27_O_18_	555.1198	555.1203	0.96	83.66		+					[18]
4	Poly-galacturonic acid	C_27_H_37_O_24_	745.1655	745.1680	3.44	74.5						+	[18]
5	Dihydroxybenzene	C_6_H_6_O_2_	109.0291	109.295	3.47	86.14	+						[4]
6	Quinic acid II	C_7_H_11_O6	191.0574	191.0561	−6.52	88.32	+				+	+	[17]
7	Caffeic acid I *	C_9_H_8_O_4_	179.0361	179.0350	−6.09	87.24			+		+	+	[4,17]
8	Chlorogenic acid *	C_16_H_18_O_9_	353.0907	353.0878	−8.34	78.95			+		+		[4]
9	Unknown	C_8_H_8_O_2_	135.0463	135.0452	−8.61	85.41							-
10	Unknown	C_8_H_7_O_2_	135.458	135.452	−4.56	93.19			+				-
11	Caffeic acid II	C_9_H_8_O_4_	179.0373	179.0350	−12.9	70.1			+				[4,17]
12	Caffeoyl-di(dihydro)caffeoylquinic acid methyl ester	C_35_H_35_O_15_	695.1865	695.1888	3.22	85.2			+				[19]
13	Caffeoyl-Hexoside	C_15_H_17_O_9_	341.0876	341.0878	0.71	86.67			+		+		[4]
14	Chlorogenic acid hexoside	C_22_H_27_O_14_	515.1441	515.1465	4.71	81.76			+				[20,21]
15	Monocaffeoylquinic acid I	C_16_H_18_O_9_	353.0899	353.0878	−6.04	81.99			+			+	[20]
16	Monocaffeoylquinic acid dimer I	C_16_H_18_O_9_	707.1885	707.1829	−7.92	70.32			+	+	+	+	[20]
17	Quinic acid III	C_7_H_12_O_6_	191.0541	191.0561	−10.31	65.63		+	+			+	[17]
18	Caffeic acid III	C_9_H_8_O_4_	179.0362	179.0350	−6.95	85.63			+			+	[17,20]
19	Unknown	C_18_H_25_O_11_	417.1445	417.1402	−10.31	56.95				+			-
20	Monocaffeoylquinic acid I	C_16_H_18_O_9_	353.0895	353.0878	−4.68	81.04				+			[4]
21	Quinic acid IV	C_7_H_12_O_6_	191.0578	191.0561	−8.99	80.7			+	+	+		[4,17]
22	Monocaffeoylquinic acid dimer I	C_16_H_18_O_9_	707.1885	707.1829	−7.92	70.32			+			+	[20]
23	Unknown	C_19_H_30_O_8_	385.1883	385.1868.	−3.82	84.23				+			-
24	Shikimic acid *	C_7_H_10_O_5_	173.0467	173.0455	−6.74	87.81			+			+	-
25	p-Coumaroylquinic acid	C_22_H_31_O_11_	471.1911	471.1872	−8.39	70.15	+		+	+		+	[19]
26	Cynaroside	C_21_H_19_O_11_	447.1034	447.0992	−9.51	81.23		+				+	[4,17,21]
27	Monocaffeoylquinic acid III	C_16_H_18_O_9_	353.0895	353.0878	−4.68	81.04			+			+	[20]
28	Monocaffeoylquinic acid IV	C_16_H_18_O_9_	353.0904	353.0878	−7.42	66.29			+				[20]
29	Monocaffeoylquinic acid V	C_16_H_18_O_9_	353.0899	353.0878	−5.79	72.14			+				[20]
30	Dicaffeoylquinic acid hexoside	C_31_H_34_O_17_	677.1726	677.1723	−0.46	80.3			+				[20]
31	Luteolin-7-O-glucoside *	C_21_H_19_O_11_	447.0943	447.0933	−2.38	78.41			+		+	+	[4,17,21]
32	Dicaffeoylquinic acid I	C_25_H_24_O_12_	515.1223	515.1195	−5.37	79.06		+	+	+	+	+	[4]
33	Dicaffeoylquinic acid III	C_18_H_28_O_17_	515.1225	515.1254	5.54	80.21			+			+	[20]
34	Unknown	C_25_H_26_O_13_	533.1317	533.1301	−2.97	75.99			+				-
35	Myricetin galloylhexoside I	C_29_H_28_O_16_	631.1309	631.1305	−0.77	79.27		+	+				[20]
36	Myricetin galloylhexoside II	C_29_H_28_O_16_	631.1309	631.1305	−0.77	79.27			+				[20]
37	Myricetin galloylhexoside III	C_29_H_28_O_16_	631.1308	631.1305	−0.54	79.09			+				[20]
38	Dicaffeoylquinic acid II	C_18_H_28_O_17_	515.1225	515.1254	5.54	80.21			+	+	+	+	[4]
39	Caffeoylquinic acid II	C_18_H_28_O_17_	515.1225	515.1195	−5.73	79.89			+			+	[20]
40	Quinic acid V	C_7_H_12_O_6_	191.0575	191.0561	−7.15	88.03		+	+		+	+	[17]
41	Monocaffeoylquinic acid II	C_16_H_18_O_9_	353.0904	353.0878	−7.42	66.29				+	+	+	[4]
42	Apigenin glucuronide	C_21_H_17_O_11_	445.0771	445.0776	1.2	79.34				+	+		[9]
43	Caffeoylquinic acid I	C_18_H_28_O_17_	515.1225	515.1195	−5.73	79.89			+	+	+	+	[4]
44	Apigeninacetyl glucoside	C_23_H_22_O_12_	489.1032	489.1038	1.31	77.72					+	+	[9]
45	4-O-Caffeoyl-5-O-[3-methoxy-3-(3,4-dihydroxyphenyl)-propionyl] quinic acid methyl ester I	C_27_H_29_O_13_	561.1613	561.1614	0.07	82.21						+	[22]
46	4-O-Caffeoyl-5-O-[3-methoxy-3-(3,4-dihydroxyphenyl) -propionyl] quinic acid methyl ester II	C_27_H_29_O_13_	561.1613	561.1614	0.05	82.45						+	[22]
47	Dicaffeoylquinic acid IV	C_18_H_28_O_17_	515.1209	515.1195	−2.74	76.53			+				[20]
48	Caffeoyl-dihydrocaffeoyl-sinapoyl quinic acid II	C_36_H_35_O_16_	723.1901	723.1931	4.08	74.8						+	[19]
49	Caffeoyl-dihydrocaffeoyl-sinapoyl quinic acid II	C_36_H_35_O_16_	723.1901	723.1931	4.08	74.8						+	[19]
50	Apigenin *	C_15_H_9_O_5_	269.0459	269.0455	−1.45	87.78					+		[21]
51	Kaempferol *	C_15_H_9_O_6_	285.0417	285.0405	−4.37	88.32	+					+	-
52	Unknown	C_35_H_55_O_13_	683.3629	683.3648	2.79	73.81				+	+		-
53	Trihydroxy-octradecenoic acid	C_18_H_33_O_5_	329.2324	329.2333	2.91	85.72					+		[19]
54	Dihydroxy-octradecenoic acid	C_18_H_31_O_4_	311.2220	311.2228	2.47	74.33					+	+	[21]
55	Unknown	C_18_H_29_O_4_	313.2422	313.2384	−11.88	55.84					+		-
56	Unknown	C_29_H_45_O_7_	564.3321	564.3093	−0.87	74.37			+				-
57	Unknown	C_15_H_22_O_3_	249.1522	249.1496	−10.25	67.16			+				-

* Confirmed by authentic chemical standards.

**Table 2:** Content of some phenolic compounds in stalks of cardoon (*Cynara cardunculus* var. *altilis* DC cv. Pieno Inerme Lucchese; PIL and *Cynara cardunculus* var. *altilis* DC cv. Plain Blanc Inerme; PBI) cultivated in the field. Mean values, mg g^−1^ DW, (±SE; *n* = 3) followed by different letters differ significantly (*p* ≤ 0.05).

Cardoon Cultivar	Treatment	^1^ Monocaffeoylquinic Acid	Caffeic Acid	^1^ Monocaffeoylquinic Acid Dimer	^1^ Dicaffeoylquinic Acid	Luteolin Glucoside
Pieno Inerme Lucchese (PIL)	Control	<LOD	<LOD	<LOD	<LOD	<LOD
	Blanching	<LOD	<LOD	<LOD	<LOD	<LOD
	Blanching+Boiling	11.54 ± 0.85	3.12 ± 0.36	43.54 ± 1.08	29.67 ± 1.15	0.05 ± 0.09
Plain Blanc Inerme (PBI)	Control	<LOD	0.09 ± 0.05	42.20 ± 0.76 a	13.30 ± 0.61 a	0.035 ± 0.06
	Blanching	<LOD	<LOD	19.12 ± 0.48 b	5.01 ± 0.50 b	<LOD
	Blanching+Boiling	0.77 ± 0.31	0.14 ± 0.08	3.26 ± 0.86 c	11.91 ± 1.39 a	0.02 ± 0.08
Significance (ANOVA)			ns	***	***	ns

<LOD: under the limit of detection; ^1^ determined with caffeic acid equivalent; significance level: *** *p* ≤ 0.001, ns: non-significant.

## Data Availability

Data sharing not applicable.
